# Identification of Bed Bugs from Comoros, Using Morphological, Matrix-Assisted Laser Desorption Ionisation Time-of-Flight Mass Spectrometry, and Molecular Biology Tools, and the Detection of Associated Bacteria

**DOI:** 10.3390/insects16020148

**Published:** 2025-02-02

**Authors:** Saidou Ahamada M’madi, Adama Zan Diarra, Jean-Michel Bérenger, Bouthaina Hasnaoui, Philippe Parola

**Affiliations:** 1 RITMES, Aix-Marseille Univ, SSA, 13005 Marseille, France; sayam1989@yahoo.fr (S.A.M.);; 2 Institut Hospitalo-Universitaire Méditerranée Infection, 19-21 Boulevard Jean Moulin, 13005 Marseille, France; 3 EMR 279 Maladies Infectieuses, Négligées et Emergentes au Sud, (MINES), IRD, 13005 Marseille, France

**Keywords:** bed bug, *Cimex hemipterus*, Comoros, MALDI-TOF MS, *Wolbachia*

## Abstract

After virtually disappearing from domestic dwellings in the Western world at the end of the Second World War, bed bugs have re-emerged in recent years. Few studies, however, have been carried out on these insects in tropical islands. In this study, we focussed on describing bed bug specimens collected from dwellings in a high-altitude village in Grande Comore, an island in the Comoros, in the Indian Ocean. We also aimed to detect the bacteria associated with them. Using MALDI-TOF MS coupled with molecular biology, we were able to confirm that the *C. hemipterus* species (the tropical bug) was the bug infesting these homes. Interestingly, the results also show that MALDI-TOF MS can differentiate between the developmental stages of bed bugs (immature and adult). Screening for bacteria was carried out using qPCR, regular PCR, and sequencing, with only *Wolbachia* DNA being found. Widespread surveys throughout the country are needed to ascertain the level of bed bug infestation, with a view to implementing appropriate control measures.

## Simple Summary

Bed bug infestations have soared worldwide in recent years and *Cimex lectularius* (common in temperate regions) and *Cimex hemipterus* (the tropical bed bug) have been identified as the primary human parasites. Despite their prevalence, information on these species remains limited, particularly in some regions. This study analysed bed bug specimens found in residential areas of the Union of the Comoros, using MALDI-TOF MS (Bruker Daltonics, Bremen, Germany) and molecular biology to confirm their morphological identification and to detect bacteria. Notably, this study is the first confirmation of *Cimex hemipterus* in the Union of the Comoros, as well as the first confirmation of the presence of a *Wolbachia* endosymbiont from Clade F.

## 1. Introduction

Bed bugs are obligate ectoparasites that feed on blood and are widespread throughout the world. Some species have been known to parasitise humans since antiquity [1]. These species belong to the family Cimicidae and are grouped into several subfamilies, namely Primicimicinae, Cimicinae, Cacodminae, Afrocimicinae, Latrocimicinae, and Haematosiphoninae [2]. Currently, *Cimex lectularius* L. 1758, the common bed bug, and *Cimex hemipterus* F. 1803, better known as the tropical bed bug, are considered the primary parasites of humans [3,4,5,6,7]. In addition to their primary host, these two species may also parasitise and survive on other mammals [8,9]. Having essentially disappeared from domestic dwellings in many Western countries in the 1950s, a strong resurgence in these ectoparasites was noted in the late 1990s, notably in England and the United States [10,11]. Since then, an upsurge in bed bug-related infestations has been reported around the world, including reports of the sympatric habitation of *C. lectularius* and *C. hemipterus* in some countries [12,13,14,15,16].

Bed bugs have been shown to harbour multiple micro-organisms, including pathogens [9,17]. Some experimental studies have aroused concern that they might transmit infectious agents. For example, laboratory studies have shown that *C. lectularius* has the ability to develop the parasite *Trypanosoma cruzi*, and *Burkholderia multivorans* is capable of infecting humans through skin lesions [18,19]. However to date, no study has confirmed the capacity of bed bugs to transmit infectious agents, and the risk of transmitted infectious diseases remains largely hypothetical [20]. That said, there is no doubt that bed bug bites on humans can cause dermatological lesions, allergies for some people, and psychological problems, such as anxiety, nervousness, and insomnia [9,21,22].

Some of the earliest evidence of bed bugs comes from surveys of Egyptian archaeological sites dating back to the time of the Pharaohs, over 3,000 years ago. Later, bed bugs were also reported on soldiers' helmets during the East African campaign in the early 1900s [1]. Currently, two species of bed bugs are known in Africa as potential parasites of humans: *C. lectularius* and *C. hemipterus* [23]. Very few studies have been conducted on bed bugs in tropical islands.

The Union of the Comoros is a country formed by three African islands (Grand Comore, Mohéli, and Anjouan) in the Indian Ocean. Geographically located north of the Mozambique Channel, in the southeast of Africa at the tip of Madagascar, the Comoros is in a strategic point facilitating trade between neighbouring countries. Through these exchanges, the country has been and continues to be exposed to the introduction of many arthropods [24,25]. However, no data on the Cimicidae fauna in the country are available.

To implement effective control strategies against arthropod pests and vectors, a reliable and rapid identification of the arthropod pest or vector is crucial. Typically, vector identification relies on species-specific morphological criteria using appropriate dichotomous keys. To be effective, however, entomological expertise is essential, as several factors related to the condition of the arthropod can influence the results, such as when part of the specimen is missing [26]. Molecular tools have also been used for reliable identification, but the relatively lengthy process, the high cost of consumables, and the absence of certain reference sequences in GenBank makes the research difficult. More generally, the high cost of equipment and the expert skills required to conduct the research work in developing nations are major hindrances.

In recent years, matrix-assisted laser desorption ionisation time-of-flight mass spectrometry (MALDI-TOF MS) has proved to be effective in identifying many arthropods [26,27,28], including bed bugs [29,30]. The objective of this study was to test the effectiveness of MALDI-TOF MS at identifying bed bugs collected from residential homes in the Comoros, as well as at identifying the bacteria associated with them.

## 2. Materials and Methods

### 2.1. Study Site and Sample Collection

**Figure 1:**
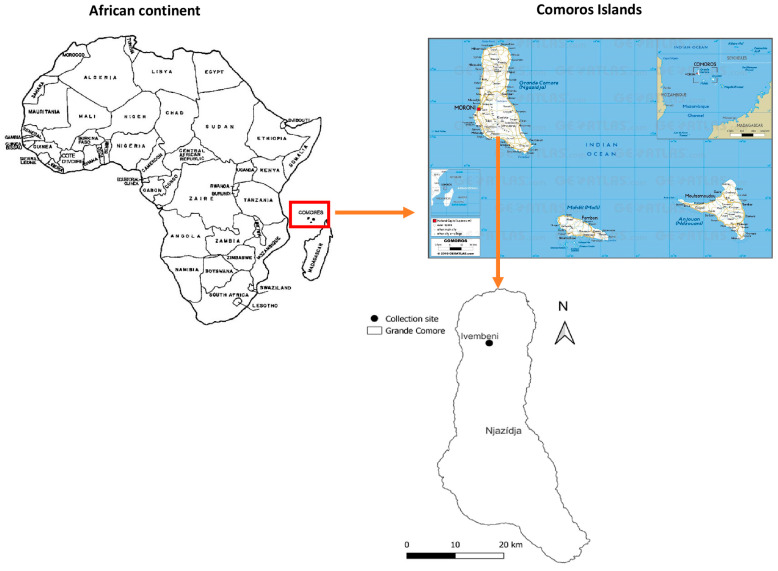
Geographical location of the study areas for the collection of bed bugs from households in the village of Ivembeni, Grande Comore.

Sampling was conducted in July and August 2022 in Ivembeni, a village in the northeast of Grande Comore at a location between 11°28′40.184″ (south latitude) and 43°19′37.991″ (east longitude) (Figure 1). The village is situated at an altitude of 822 m. It is an agricultural area that experiences significant rainfall, with an average annual precipitation of 1595.1 mm and an average temperature of 26.6 °C. Many Comorians consider Ivembeni to be a very friendly and sociable village, and as a result it has become a very lively and eventful place. Investigations were carried out in three homes located in the same area. Sampling was carried out with the verbal agreement of the owners of the homes, as well as the consent of those occupying the various rooms. Bed bugs were collected from bedding. The samples of bed bugs collected from households were preserved in 70% ethanol and transported to the laboratory of the Institut Hospitalo-Universitaire (IHU) Méditerranée Infection in Marseille, France, for further study, following approval by the French Ministry of Agriculture (reference ER40-2022).

### 2.2. Morphological Identification of Bed Bugs and Dissection

Once at the IHU laboratory, the adult specimens were identified morphologically using the discriminating criteria of the taxonomic identification key previously established [8]. A stereomicroscope with ×56 magnification (Zeiss Axio Zoom.V16, Zeiss, Marly-le-Roi, France) was used to determine the sex and identify the different developmental stages of the specimens. The bed bugs were then dried overnight in a dry water bath at 37 °C. Each specimen was then dissected using a sterile surgical blade. The heads of adult specimens and the cephalothorax of immature specimens were removed and placed in individual Eppendorf tubes for MALDI-TOF MS analyses. The remainder of the body was then divided lengthwise into two equal parts, one being used for molecular biology analyses and the other frozen at −20 °C for later study.

### 2.3. DNA Extraction and Molecular Identification of Bed Bugs

The longitudinal halves of the nine selected specimens were subjected to molecular analysis. The half of the body that was stored at −20 °C was transferred into a 1.5 µL Eppendorf tube containing 180 µL of G20 lysis buffer and 20 µL of Proteinase K (Qiagen, Hilden, Germany). All were incubated overnight at 56 °C. DNA extraction was then performed individually using the NucleoMag pathogen fast rev05 protocol (MACHEREY-NAGEL, Düren, Germany), following the manufacturer’s recommendations. DNA from nine bed bug specimens was submitted to standard PCR in an automated thermal cycle (Applied Biosystems, Foster City, CA, USA) using the 18S rDNA primer amplifying a 951 base-pair fragment. For molecular analyses, we used the pair 18S3f_5′-GAG TCT CGT TCG TTA TCG GA-3′ and 18SBi_5′-GTT CGA TTC CGG AGA GGG A-3′ as the primer sequence, as previously described [31]. As a positive control, we used DNA obtained from a *C. hemipterus* specimen from our laboratory colonies, strains of which were obtained from the Cimex Store (Chepstow, UK) and have been maintained in our laboratory since 2018. The results obtained after sequencing with 18S rDNA were assembled and analysed using ChromasPro software (version 1.7.7) (Technelysium Pty. Ltd., Tewantin, Australia). The corrected sequences were then compared against the NCBI GenBank database (https://blast.ncbi.nlm.nih.gov/Blast.cgi, “URL (accessed on 14 August 2023)”) using a BLAST search to confirm species identity.

### 2.4. Sample Preparations for MALDI-TOF MS Analyses

The heads of adults and heads and thoraxes of immatures were incubated at 37 °C overnight and individually homogenised in 15 μL of an extraction solution composed of 70% formic acid and 50% acetonitrile, with glass beads (1.0 mm diameter, BioSpec Products) using a TissueLyser (Qiagen, Hilden, Germany) with set parameters of three one-minute cycles at a frequency of 30 Hertz. The homogenised samples were then centrifuged at 2000× *g* for one minute, with 1 μL of the supernatant from each sample deposited on the quadruple MALDI-TOF MS steel target plate (Bruker Daltonics, Wissembourg, France). After drying, 1 µL of a CHCA matrix suspension composed of saturated α-cyano-4-hydroxycinnamic acid (Sigma-Aldrich, Lyon, France), acetonitrile, trifluoroacetic acid (Sigma-Aldrich, Dorset, UK), and HPLC-grade water was added. Homogenates from the head of the *C. hemipterus* laboratory sample were used as a positive control.

### 2.5. MALDI-TOF MS Parameters

Protein mass profiles were generated using a Microflex LT MAL-DI-TOF MS (Bruker Daltonics, Germany), with the linear mode detection of positive ions at a laser frequency of 50 Hz over a mass range of 2 at 20 kDa. The accelerating voltage was 20 kV and the extraction delay time was 200 ns [32]. Each spectrum corresponded to the ions generated from 240 laser shots carried out in six regions at the same point and acquired automatically using the AutoXecute method in FlexControl v2.4 software (Bruker Daltonics, Bremen, Germany).

### 2.6. Spectral Analysis, Database Creation, and Blind Test

MS spectral profiles were initially inspected visually using flexAnalysis v3.3 software (Bruker Daltonics, Bremen, Germany). The MS spectra were then exported to ClinProTools v2.2 and MALDI-Biotyper v3.0 (Bruker Daltonics) for data processing, including smoothing, baseline subtraction, and peak detection. Reproducibility, the absence of background noise, and a peak intensity > 3000 arbitrary units were used as the criteria to assess the quality of the spectra. Only MS spectra meeting these criteria were selected for further analysis. The reproducibility of the MS spectra was assessed by comparing the master spectrum profiles (MSPs) obtained from the four spots of each sample with MALDI-Biotyper v3.0 software (Bruker Daltonics). To select the reference MS spectra to be included in the in-house database, the reproducibility and specificity of the spectra were evaluated using unsupervised statistical tests, including principal component analysis (PCA) and cluster analysis (MSP dendrogram), performed with ClinProTools v2.2 and MALDI-Biotyper v3.0 software.

Spectra from six bed bug specimens (three adults and three immature specimens), identified using molecular biology, were added to our in-house database containing 2232 spectra from several arthropod species, including the spectra obtained from the heads of adult *C. lectularius* from the field, the French strain specimen, *C. hemipterus* from various countries (Senegal and Cameroon), *C. hirundinis* from France [29,30,33,34], immature *C. hemipterus* [35], and the heads and thoraxes from immature *C. hirundinis* (France) and *C. hemipterus* (Cameroon and laboratory strain) [29,36]. Reference MS spectra were obtained using an unbiased algorithm and information on the peak position, intensity, and frequency. Blind tests were performed on the updated database using the remaining MS spectra. The reliability of species identification was estimated using log score values (LSVs) obtained through MALDI-Biotyper software v3.0, ranging from 0 to 3. An LSV was generated for each spectrum of the samples tested. We used R-studio v4.0.5 software to produce boxplots representative of the LSV scores to identify specimens.

### 2.7. Detection of Bacteria Associated with Bed Bugs

To detect the bacteria associated with our bed bug samples, quantitative PCR (qPCR) using primers and probes targeting various bacterial pathogens, including the Anaplasmataceae family, *Rickettsia* spp., *Borrelia* spp., *Coxiella burnetii*, and *Bartonella* spp., was performed on the DNA extract obtained [33,37]. qPCR was carried out in a CFX96 real-time system (Bio-Rad Laboratories, Foster City, CA, USA). The reaction mixture contained 5 µL of DNA template and 15 µL of reaction mixture, as previously described [36]. Only samples with a CT value below 36 were considered to be positive [33]. Samples that were found to be positive for Anaplasmataceae were then subjected to qPCR specifically for the detection of *Wolbachia*. On each qPCR, positive controls included DNA from *Borrelia crocidurae*, *Anaplasma phagocytophilum*, *Coxiella burnetii*, *Rickettsia montanensis*, and *Bartonella elizabethae*, and negative controls (DNA from *Rh. sanguineus* s.l. from our laboratory, which are known to be free from these bacteria) were used, as described previously [35]. Ten randomly selected *Wolbachia*-positive DNA samples were subjected to standard PCR targeting 560 base-pair fragments of the ftsZ gene. All the primers and probes used in this study are summarised in Table 1. The sequences obtained were assembled and then compared against GenBank by the NCBI BLAST search (https://blast.ncbi.nlm.nih.gov/Blast.cgi, “URL (accessed on 26 September 2024)”). MEGA version 7.0 software was used to perform our sequence alignments and build the phylogenetic tree with 1000 bootstrap replications for the ftsZ gene.

## 3. Results

### 3.1. Morphological and Molecular Identification of Bed Bugs

**Figure 2:**
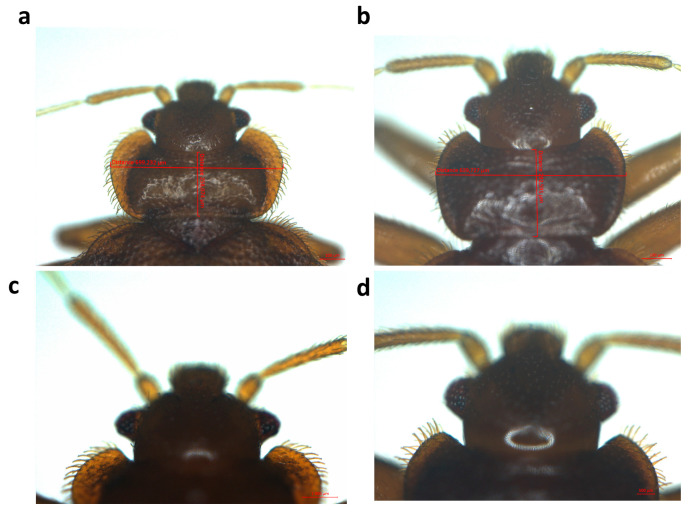
Illustrative images of two species of adult bed bugs, *Cimex lectularius* (laboratory specimen) and *Cimex hemipterus* (this study), taken using the Zeiss Axio Zoom V16 stereomicroscope (Zeiss, Marly-le-Roi, France). (**a**,**c**) Pronotum and pronotal bristles of *C. lectularius*; (**b**,**d**) pronotum and pronotal bristles of *C. hemipterus*. µm: Micrometre.

A total of 168 bed bugs were collected from the three homes that were investigated. Sixty-eight of them (40%) were found to be adults and 100 (60%) were found to be nymphs. Of the 68 adults, 43 (63%) were males and 25 (37%) were females. Only two of the dwellings were infested, and no traces of bed bugs were found in the third. In the two infested homes, bed bugs were discovered in the mattresses, along the edges of the beds, and within the walls (Figure 1). All the bed bugs collected were identified morphologically as *C. hemipterus*, taking into account the size of pronotum, the shape of pronotum hairs, and the size of heads (Figure 2) [8]. Nine randomly chosen specimens were submitted to standard PCR and sequencing using the 18S rDNA gene. All obtained sequences revealed a similarity between 99.96% and 100% with the *C. hemipterus* sequence from Senegal (GenBank accession number: MN056507).

### 3.2. MALDI-TOF MS Identification of Bed Bugs

**Figure 3:**
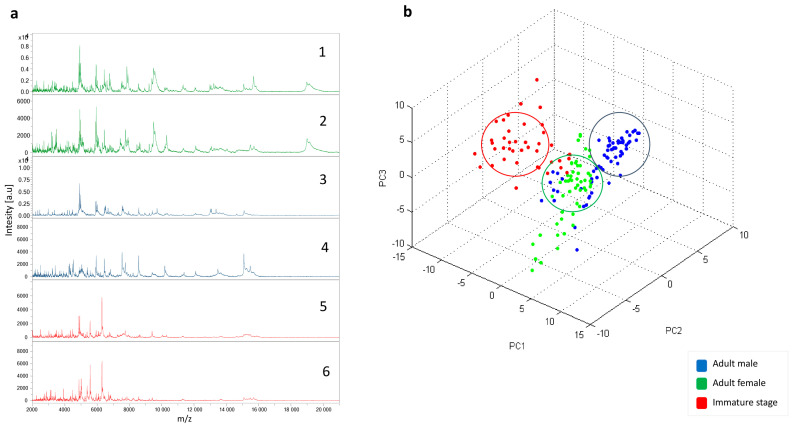
Comparison of protein profiles of *Cimex hemipterus* specimens using ClinProTools software and principal component analysis (PCA) showing the dimensional clustering of specimens based on their MALDI-TOF MS profiles. (**a**) Spectra representative of the different stages of development. (1 and 2) Spectra of adult male specimens; (3 and 4) spectra of adult females; (5 and 6) representative spectra of immature stages. (**b**) Dimensional distribution of specimens based on their MS profiles. au, Arbitrary unit; m/z, mass/charge ratio.

**Figure 4:**
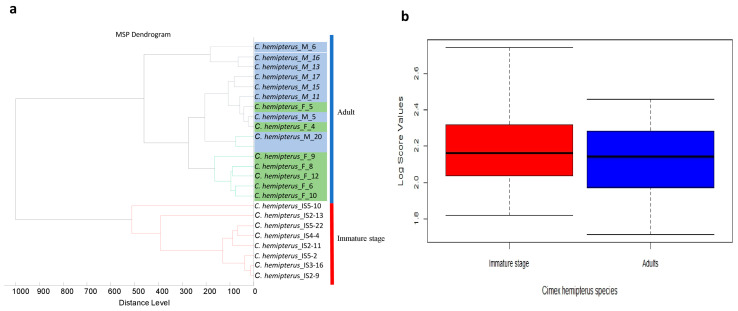
(**a**) Clustering dendrogram created from MS spectra of adult *C. hemipterus* and their nymphs. Cluster analysis was performed using Biotyper software v.3.0. (**b**) Graphical representation of the distribution of LSV scores for identifying the different stages of development. In red, the immature stage, and in blue, adults. The green and blue boxes represent the grouping of adult individuals according to sex. Abbreviations: F, female; M, male.

A total of 145 specimens, including 58 adults and 87 nymphs, were subjected to MALDI-TOF MS analysis. The analysis of the MS spectra obtained using flexAnalysis v.3.3 software revealed that 132/145 (91%) of the spectra were of high intensity and visually reproducible, with no background noise (Figure 3a). Applying PCA to the spectra grouped between the adult and immature stages showed that each group was clustered in a distinct branch, suggesting the specificity of the MS spectra between the immature and adult stages (Figure 3b). This clustering was confirmed by cluster analysis (dendrogram) showing very distinct branch clustering of these two groups. In contrast, within the adult stages, an overlap between males and females was observed (Figure 4a). Six MALDI-TOF MS spectra were added to our reference database. Querying the remaining 126 MALDI-TOF MS spectra against the enhanced reference spectra database revealed 100% correct identification at the species level with scores ranging from 1.81 to 2.74 (mean of 2.20 and median of 2.21) and from 1.71 to 2.45 (mean of 2.10 and median 2.13) for the immature stages and adult stages, respectively. Of the 126 MS spectra of correctly identified specimens, 123 (97.6%) had LSVs scores ≥1.8. Interestingly, 74/78 (94.87%) and 41/48 (85.41) correct identifications were recorded for the immature and adult stages, respectively. The list of randomly selected bed bug spectra we added to our in-house database can be found here: https://doi.org/10.35081/s706-y441. All log score values obtained for adults and nymphs are shown in Figure 4b and Table 2.

### 3.3. Detection of Bacteria

**Figure 5:**
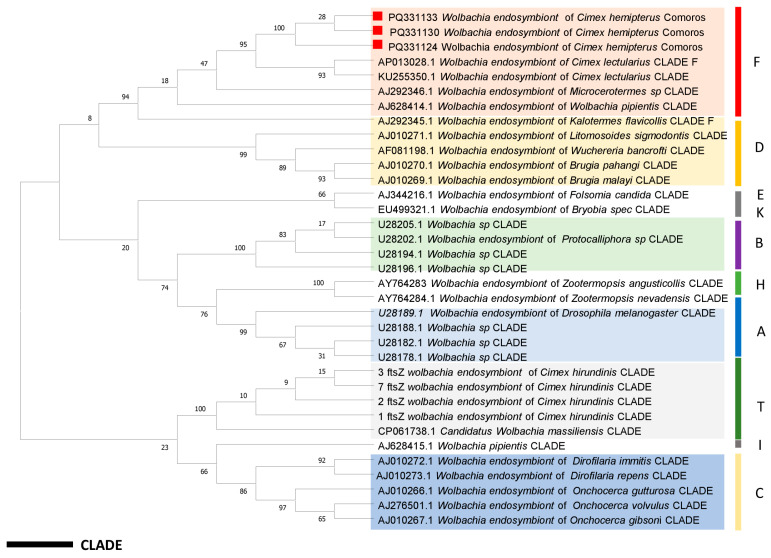
Phylogenetic tree (neighbour membership, 1000 bootstraps) based on the *Wolbachia* sequences of the genes encoding the ftsZ protein (560 bp), indicated by the names of the host species and the clade to which they belong. Sequences from the Comoros samples are shown in red, and the other colours correspond to GenBank sequences.

The DNA of 145 *C. hemipterus* specimens was used to detect bacteria by qPCR. Only 67/145 samples (46%) tested positive for bacteria of the Anaplasmataceae family. All samples that were positive for bacteria of the Anaplasmataceae family tested with the 23S rRNA gene were found to be positive for *Wolbachia* using specific qPCR with the 16S rRNA gene. For the other bacteria tested, all samples were negative. The ten sequences obtained from the *Wolbachia*-positive samples with FtsZ showed 100% identity to the corresponding sequence in the complete genome of the *Wolbachia* endosymbiont isolated from *C. lectularius* (GenBank: AP013028) from Japan and were deposited under accession numbers ranging from PQ381124 to PQ381133. Using the maximum likelihood method, phylogenetic analyses revealed that our sequences belonged to supergroup F (Figure 5).

## 4. Discussion

As elsewhere in the world, a resurgence of bed bug infestations has been reported in Africa, in refugee camps, shelters, and households [44,45,46]. The end of the use of dichlorodiphenyltrichloroethane (DDT) and other organochlorines, and the emergence of resistance to insecticides currently used in Africa may be drivers of the global resurgence of bed bugs, which has led to a rapid and wide spread of bed bugs across the continent [46,47,48,49,50].

In the Comoros, and more specifically in Grande Comore, information on bed bug infestations is very limited. The first documentation on bed bugs was a report published in 1978, in which the authors reported the presence of the *C. lectularius* species in Mayotte (an island in the Comoros archipelago that has remained a French territory) and the abundance of bed bugs on the other islands, but with no further information on the species present [51]. This study, therefore, constitutes the first survey of bed bug infestations on Grande Comore, focussing on a very busy village at a relatively high altitude (Ivembeni). In the literature, several studies have reported that the increase in human migration, as well as international trade, has passively contributed to the global resurgence of bed bugs, and that large cities frequented by tourists and traders are the most exposed to this scourge [7,44,52]. Bed bugs hide in cracks, furniture, sleeping areas (corners of mattresses, mosquito nets, sheets, and box springs), cushions, and sofas, as well as in papers [6,13,33]. They usually bite sleeping humans to consume their blood meal.

In this study, based on the morphological criteria of adult specimens, all bed bug species were identified as *C. hemipterus*. The difficulty of definitively identifying immature stages lies in the absence of an operational identification key suitable for distinguishing these stages, making their classification highly challenging. The published studies, based on the morphometry of the head, antennae, and pronotum of immature stages, have produced an imperfect classification [52,53]. Although molecular biology can be used effectively and with certainty to identify arthropods, including bed bugs [29,34], the relatively long handling time can be a major drawback to their usefulness, not to mention the cost of the consumables they require. In this study, the cost of molecular tools for a small number of samples was not a concern. However, we employed these tools to ensure the definitive identification of the bed bugs, the spectra of which were introduced into the MALDI-TOF MS arthropod database.

In recent years, the success of MALDI-TOF mass spectrometry in the field of clinical microbiology has led to the use of this tool in entomology for the identification of the arthropods themselves, the nature of their blood meals, and the determination of their infectious status [26]. However, during sample preparation, several factors, including the method and duration of sample storage, the amount of mixing buffer, and sample grinding parameters, can lead to changes in the expected MALDI-TOF MS results.

In this study, the use of the heads and thoraxes for immature bed bugs and the heads of adults, stored in 70% alcohol, enabled us to obtain spectra of good quality, which were reproducible and specific from one stage of development to another. Of the 145 samples processed, 132/145 (91%) revealed good-quality and reproducible spectra, in contrast to the previously reported results where the percentages were low [35,36]. This difference in percentage, despite the samples being stored in the same way (70% ethanol), can be attributed to the quantity of the homogenisation mixture, which was 15 µL for all samples. This volume was sufficient to achieve a protein concentration in each homogenate that allowed peak intensification. These results clearly highlight the importance of carefully selecting the protocol used during sample preparation.

To ensure correct and reliable identification and to avoid any errors, especially concerning closely related species, updating the reference database remains a crucial step. This is why it is important to use expertise in entomology and molecular biology to identify the species morphologically and confirm by molecular biology before adding any reference spectra. In this study, the spectra of six specimens, confirmed by molecular biology, were added to the reference database, based on three spectra for each stage of development. All the spectra compared with the updated database were correctly matched with the correct species. When it came to discriminating between male and female adults, we observed an overlap in the dendrogram in the branches between males and females, as well as at the level of the PCA, where an inappropriate distribution was observed. These results are comparable to those recently reported, showing no difference between the spectra of male and female bed bugs [34,36]. This non-discrimination between adult male and female bed bugs can be explained by environmental factors and their microbiotic constituents, given that our specimens are wild species, in contrast to the study by Benkacimi et al., which used specimens from breeding colonies [30]. Indeed, different biotope conditions play a major role in the protein composition of arthropods. It has been reported that the digestive tract contents of arthropods can have a negative impact on MALDI-TOF MS spectra by alternating the MALDI-TOF MS profiles of samples [54]. This phenomenon has been linked to the microbiotic composition of the species, more specifically the infectious status of the species and how its immune system can produce additional proteins capable of being detected in protein profiles following infection, as previously reported in mosquitoes and ticks [55,56,57].

Regarding the originality of this research conducted, it should be stressed that this is not an iteration of the few studies in this field previously published, but simply in a different region. This study employed this valuable proteomics technique to identify bed bugs and was shown to differentiate between adult and immature stages with considerable success. It offers greater insight into the potential application of MALDI-TOF MS in entomological research. Additionally, this research is integrated into the training programmes we conduct to enable young people from the Global South to access and master advanced technologies.

In this study, we limited the detection of bacteria to those that fall within the expertise of our laboratory. No micro-organisms other than *Wolbachia* were detected. These bacteria are well known as arthropod endosymbionts, and their role in the reproduction and development of bed bugs, as well as in the synthesis of vitamin B, an important vitamin acting as a coenzyme in the metabolic processes of detoxification of heme and iron from the digestion of blood meals, has been reported [58]. In total, a 46% carriage rate of *Wolbachia* endosymbionts was reported in this study. This rate appears to be lower than that reported in other studies, where field-caught bed bugs were 100% positive for *Wolbachia*. Thus, *Wolbachia* has been described as an obligate bacterium in *C. lectularius*. Transovarial transmission has been reported. Such an association suggests that all individuals in some *C. lectularius* populations would be infected with *Wolbachia* [59]. However, other studies have revealed variations in the frequency and relative abundance of *Wolbachia* in field samples of bed bugs [36,60,61]. This abundance of *Wolbachia* in bed bugs seems to be influenced by various factors, including the developmental stage, the intermoult stage, and the blood-feeding status of species [59]. It has also been reported that prolonged starvation in arthropods can have a negative impact on *Wolbachia* titration and can even lead to its elimination [62]. This may also explain the low *Wolbachia* carriage rate in our study, given that our samples were mainly collected from a room reserved for guests with a limited number of visitors and therefore sources of blood meals. Finally, all *Wolbachia* reported in this study were identified as belonging to *Wolbachia* supergroup F, which has already been described as a specific symbiont bacteria infecting arthropods and nematodes [60].

## 5. Conclusions

Entomological monitoring of bed bugs is becoming a necessity to combat the rapid spread of these species, which is currently being reported in different parts of the world. This study constitutes the first concrete proof of the presence of the *C. hemipterus* species on Grande Comore, along with its associated bacterium, a *Wolbachia* endosymbiont. To combat the rapid spread of these blood-feeding pests, residents need to be made more aware of the various control techniques. The priority is to advise residents to use mechanical controls, such as hoovers, steam, and diatomaceous earth. A national survey needs to be carried out to assess the actual level of bed bug infestations.

Our results confirm that MALDI-TOF MS can be used to identify bed bug species. Setting up a MALDI-TOF MS platform in the Comoros islands would be a crucial step forward, not only because it could be used in entomological surveillance for the identification of arthropod vectors/pests, but also because it could be beneficial to other areas of research, such as parasitology and microbiology, as in the case of Senegal [63]. Ultimately, our findings contribute to the growing body of literature on innovative proteomic methods for insect identification and highlight the importance of accurate species-level identification in pest management and public health. Although species identification using the protein profiling methodology is now quite convincing, more work is needed to test whether MALDI-TOF would be able to distinguish between the immature stages of various *Cimex* spp., as well as the different life stages within each species.

## Figures and Tables

**Table 1:** Primers used to detect bacteria in this study for quantitative real-time reactions (qPCRs) and standard polymerase chain reactions (PCRs).

Assay Specificity	Targeted Gene	Primers (5′-3′) and Probes	Reference
*Wolbachia* spp.	16S	f_TGGAACTGAGATACGGTCCAG r-GCACGGAGTTAGCCAGGACT p_FAM-AATATTGGACAATGGGCGAA	[38]
	FtsZ *	f_GGRATGGGTGGTGGYACTGG r_GCATCAACCTCAAAYARAGTCAT	[38]
Anaplasmataceae	23S	f_TGACAGCGTACCTTTTGCAT r_GTAACAGGTTCGGTCCTCCA p_6FAM-GGATTAGACCCGAAACCAAG	[39]
*Rickettsia* spp.	gltA	f_GTGAATGAAAGATTACACTATTTAT r_GTATCTTAGCAATCATTCTAATAGC p_6FAM-CTATTATGCTTGCGGCTGTCGGTTC	[40]
*Borrelia* spp.	ITS4	f_GGCTTCGGGTCTACCACATCTA r_CCGGGAGGGGAGTGAAATAG p_6FAM-TGCAAAAGGCACGCCATCACC	[41]
*Bartonella* spp.	ITS2	f_GATGCCGGGGAAGGTTTTC r_GCCTGGGAGGACTTGAACCT p_GCGCGCGCTTGATAAGCGTG	[42]
*Coxiella burnetii*	IS1111A	f_CAAGAAACGTATCGCTGTGGC r_CACAGAGCCACCGTATGAATC 6FAM-CCGAGTTCGAAACAATGAGGGCTG	[43]

* Primer for standard PCR.

**Table 2:** MALDI-TOF MS identification of adults and immature specimens of *C. hemipterus*.

Stage of Development	Morphological ID	No. Tested	18S Molecular Identification	Good Spectra	Reference Spectra	Blind Test	MALDI Identification with the Right Species	Score Range
Adults	*C. hemipterus*	58	*C. hemipterus* (3) *100%*	51/58	3	48	48/48 (100%)	1.71–2.45
Immature stage	-	87	*C. hemipterus* (3) 100%	81/87	3	78	78/78 (100%)	1.81–2.74
Total	-	145	6	132	6	126	126/126 (100%)	-

## Data Availability

The data presented in this study (reference spectra) and (sequences) can be consulted free of charge at DOI: https://doi.org/10.35081/s706-y441, “URL (accessed on 26 September 2024)”, and in GenBank (National Centre for Biotechnology Information, NCBI) under the following accession numbers: PQ381124 to PQ381133.
